# iMFP-LG: Identify Novel Multi-functional Peptides Using Protein Language Models and Graph-based Deep Learning

**DOI:** 10.1093/gpbjnl/qzae084

**Published:** 2024-11-25

**Authors:** Jiawei Luo, Kejuan Zhao, Junjie Chen, Caihua Yang, Fuchuan Qu, Yumeng Liu, Xiaopeng Jin, Ke Yan, Yang Zhang, Bin Liu

**Affiliations:** School of Computer Science and Technology, Harbin Institute of Technology, Shenzhen 518055, China; School of Science, Harbin Institute of Technology, Shenzhen 518055, China; School of Computer Science and Technology, Harbin Institute of Technology, Shenzhen 518055, China; School of Computer Science and Technology, Harbin Institute of Technology, Shenzhen 518055, China; School of Computer Science and Technology, Harbin Institute of Technology, Shenzhen 518055, China; College of Big Data and Internet, Shenzhen Technology University, Shenzhen 518055, China; College of Big Data and Internet, Shenzhen Technology University, Shenzhen 518055, China; School of Computer Science and Technology, Beijing Institute of Technology, Beijing 10081, China; School of Science, Harbin Institute of Technology, Shenzhen 518055, China; School of Computer Science and Technology, Beijing Institute of Technology, Beijing 10081, China; Advanced Research Institute of Multidisciplinary Science, Beijing Institute of Technology, Beijing 10081, China

**Keywords:** Multi-functional peptide discovery, Protein language model, Graph attention network, Therapeutic peptide screening, Deep learning

## Abstract

Functional peptides are short amino acid fragments that have a wide range of beneficial functions for living organisms. The majority of previous studies have focused on mono-functional peptides, but an increasing number of multi-functional peptides have been discovered. Although there have been enormous experimental efforts to assay multi-functional peptides, only a small portion of millions of known peptides has been explored. The development of effective and accurate techniques for identifying multi-functional peptides can facilitate their discovery and mechanistic understanding. In this study, we presented iMFP-LG, a method for multi-functional peptide identification based on protein language models (pLMs) and graph attention networks (GATs). Our comparative analyses demonstrated that iMFP-LG outperformed the state-of-the-art methods in identifying both multi-functional bioactive peptides and multi-functional therapeutic peptides. The interpretability of iMFP-LG was also illustrated by visualizing attention patterns in pLMs and GATs. Regarding the outstanding performance of iMFP-LG on the identification of multi-functional peptides, we employed iMFP-LG to screen novel peptides with both anti-microbial and anti-cancer functions from millions of known peptides in the UniRef90 database. As a result, eight candidate peptides were identified, among which one candidate was validated to process both anti-bacterial and anti-cancer properties through molecular structure alignment and biological experiments. We anticipate that iMFP-LG can assist in the discovery of multi-functional peptides and contribute to the advancement of peptide drug design.

## Introduction

Functional peptides are short amino acid fragments, usually ≤ 50 amino acids, which play an important role in the regulation of a variety of biological functions, such as regulating hormones, neurotransmitters, and growth factors [1,2]. Because of their excellent selectivity, effectiveness, comparative safety, and good tolerability in biological activities, functional peptides have attracted tremendous attention in medicine [3–5]. Up to now, peptides with a wide range of functions have been discovered [6,7], including anti-microbial peptides (AMPs) and anti-cancer peptides (ACPs). Notably, an increasing number of peptides have been demonstrated to have multiple functions. For instance, some AMPs show lethal effects on cancer cells [8]. Effective and accurate techniques for identifying multi-functional peptides can facilitate their discovery and mechanistic understanding. Unfortunately, biological experiments for studying peptide functions are time-consuming and expensive in both labor and materials.

Currently, computational approaches have achieved remarkable success in the discovery of peptide functions [5,9–12]. With the development of machine learning techniques, the methodologies of peptide discovery have gone through three stages: conventional machine learning-based methods, deep learning-based methods, and pre-trained protein language model (pLM)-based methods. Conventional machine learning-based methods (*e.g.*, AVPpred [13], PredAPP [14], AIPpred [15], THPep [16], and AntiCP 2.0 [17]) identify peptides by employing Support Vector Machine (SVM) and Random Forest (RF) algorithms based on various feature engineering techniques, including Position-Specific Scoring Matrix (PSSM) [18], physicochemical properties [19], and pseudo amino acid composition (PseAAC) [20]. Despite their effectiveness, conventional machine learning-based methods are often hard to generalize to other peptide datasets. In contrast to the hand-crafted features in conventional machine learning-based methods, deep learning-based methods (*e.g.*, DeepACP [21], Su et al. [22], Veltri et al. [23], and Ma et al. [24]) automatically extract features by employing various deep learning architectures, including Convolutional Neural Network (CNN), Long Short Term Memory (LSTM), and their combination, for distinguishing functional peptides. Some researchers have combined various feature engineering techniques with deep learning methods to construct powerful predictors (*e.g.*, iACP-DRLF [25], ACP-MHCNN [26], ITP-Pred [27], MLACP 2.0 [28], and ACP-2DCNN [29]). However, due to the small datasets of functional peptides, these supervised deep learning-based methods encounter the challenge of learning robust peptide representation. Recently, pre-trained language models have emerged as a novel powerful paradigm in the field of natural language processing (NLP) [30,31]. They are typically initially trained on extensive datasets in a self-supervised manner and are subsequently leveraged for a myriad of downstream tasks. pLMs have also been proposed and applied to peptide identification [32], such as anti-bacterial peptides (ABPs) [33], AMPs [24], signal peptides [34], anti-hypertensive peptides (AHPs) [35], and bitter peptides [36]. However, these studies focus on mono-functional peptide prediction.

In contrast to the discovery of mono-functional peptides, multi-functional peptide identification is a multi-label classification task, which assigns a set of relevant functional labels to a peptide simultaneously. Multi-label classification is more complex due to the challenges in capturing hidden connections of labels and resolving data imbalances. Several studies have made a difficult endeavor in the discovery of multi-functional peptides. Tang et al. [37] and Li et al. [38] identified multi-functional peptides by using a multi-label deep learning method, which combines CNN and recurrent neural network (RNN) to extract peptide features and assigns function labels separately. PrMFTP [39] improved the performance of multi-functional therapeutic peptide identification by employing a multi-scale CNN and an attention-based bidirectional long short-term memory (BiLSTM). Since the number of multi-functional peptides is fewer than that of mono-functional peptides, the training datasets are extremely imbalanced. ETFC [40] utilized a text CNN combined with a multi-label focal dice loss function to solve the inherent imbalance problem in the multi-functional peptide prediction. Although some training tricks have effectively mitigated the effect of imbalanced data, existing methods still lack generalization to learn comprehensive peptide representations and have high false positive rates. In addition, they predicted all function labels independently without considering their correlations. Pre-trained pLMs and graph attention networks (GATs) offer solutions to these problems. The pLMs pre-trained on millions of protein sequences [41] can capture long-range dependencies of amino acid residues [42–44]. GATs have the ability to capture complex relationships by computing attention coefficients between different objects [45]. The advantages of both pLMs and GATs can provide helpful insights for the discovery of multi-functional peptides.

In this study, we developed a method, iMFP-LG, for discovering multi-functional peptides based on pLMs and GATs. To the best of our knowledge, iMFP-LG is the first approach that considers the associations between function labels to identify multi-functional peptides by converting the multi-label problem to the graph node classification. The computational results showed that iMFP-LG outperformed the state-of-the-art methods on both multi-functional bioactive peptide (MFBP) and multi-functional therapeutic peptide (MFTP) datasets. iMFP-LG is also interpretable by visualizing the distribution of peptide representations, motif patterns obtained by the pLM, and function relationships captured by the GAT. We employed the iMFP-LG model to establish a robust peptide discovery pipeline. Through this pipeline, eight novel candidate multi-functional peptides were screened out with high confidence from the UniRef90 database, which is an extensive collection of millions of known peptides. Further biological experiments showed that one of the candidates had remarkable bioactivities in terms of anti-microbial and anti-cancer functions. These results demonstrate the capability of iMFP-LG for the discovery of novel multi-functional peptides.

## Results and discussion

### Overview of the proposed method iMFP-LG

**Figure 1:**
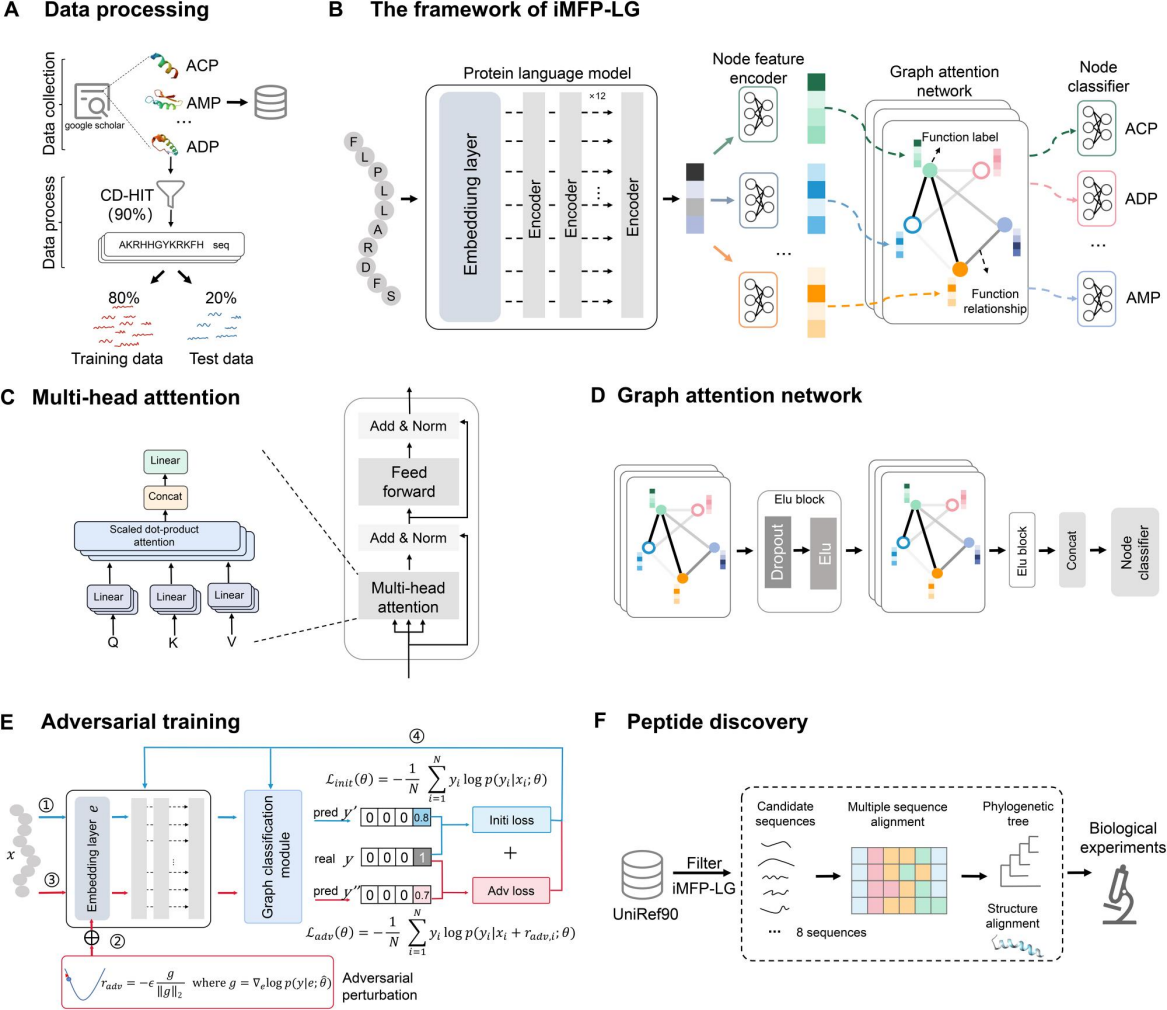
Illustration of the proposed iMFP-LG **A**. Collection and processing of multi-functional peptide datasets. **B**. The framework of iMFP-LG, including a peptide representation module and a graph classification module. The peptide representation module employs a protein language model to extract high-quality peptide representations from primary sequences. The graph classification module is composed of a node feature encoder, a graph attention network, and a node classifier to learn the correlation of function labels. **C**. The multi-head attention mechanism of an encoder layer in the protein language model. **D**. Two stacked graph layers in the graph attention network. **E**. The procedure of adversarial training. **F**. The pipeline of peptide discovery by iMFP-LG.

The architecture of iMFP-LG consists of two modules: a peptide representation module and a graph classification module (**Figure 1**). Within the peptide representation module, the pLM is responsible for acquiring high-quality peptide representations through a multi-head self-attention mechanism. In the graph classification module, the GAT is employed to capture the interrelationships among various function labels. The nodes in the graph are function labels and the edges are the correlations between function labels. The node features are first generated by the pLM according to the input peptide sequences, while edge weights are learned by the GAT. All node features are further updated in the GAT to integrate complex relationships between peptide function labels according to the corresponding edge weights. At last, the multi-functions of peptides are determined by a set of node binary classifiers based on the updated node features. In addition, adversarial training is used to improve model robustness and generalization ability. To discover novel multi-functional peptides, we established a robust pipeline based on the trained iMFP-LG model. The architecture of iMFP-LG and the adversarial training are introduced as follows.

### Graph node classification framework improves discovery of multi-functional peptides

The discovery of multi-functional peptides is a multi-label classification task. To capture the complex relationships among multi-functions, we proposed a graph node classification framework. In this section, we evaluated the effectiveness of graph node classification framework on several widely used sequence feature extraction methods, including four sequence composition features (CFs) extracted by iFeatureOmega [46], *i.e.*, amino acid composition (AAC), pseudo-amino acid composition (PAAC) [20], Distance-Pairs (DP) [47], and Composition-Transition-Distribution (CTDD) [48], three deep learning-based methods (*i.e.*, CNN, RNN, and CNN+BiLSTM), and a pLM-based method (*i.e.*, TAPE [49]).

**Figure 2:**
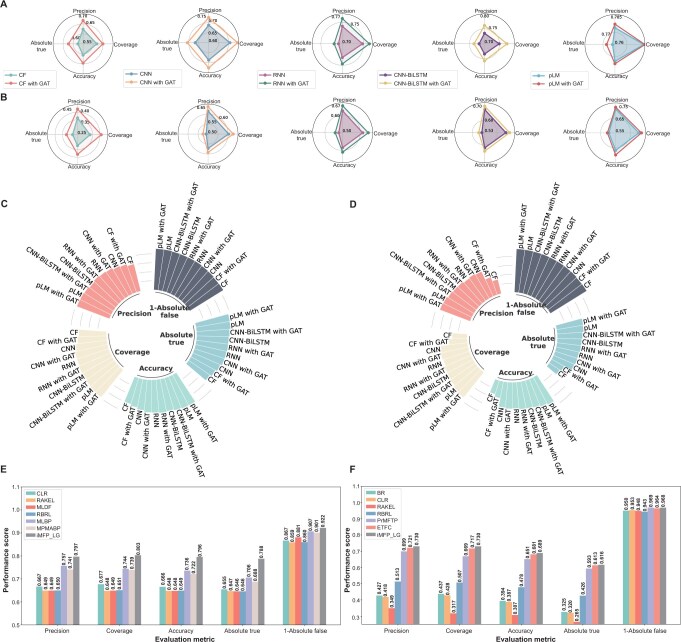
Performance comparison of different methods for multi-functional peptide identification **A**. and **B**. Effect of GAT on different feature extraction methods on the MFBP (A) and MFTP (B) datasets. **C**. and **D**. Performance comparison of different feature extraction methods on the MFBP (C) and MFTP (D) datasets. **E**. and **F**. Performance comparison of our proposed method iMFP-LG and the state-of-the-art methods on the MFBP (E) and MFTP (F) datasets. GAT, graph attention network; pLM, protein language model; CF, composition feature; MFBP, multi-functional bioactive peptide; MFTP, multi-functional therapeutic peptide; CNN, convolutional neural network; RNN, recurrent neural network; BiLSTM, bidirectional long short-term memory.

We first evaluated the performance of abovementioned feature extraction methods with or without GAT on the MFBP (**Figure 2**A) and MFTP (Figure 2B) datasets in terms of precision, coverage, accuracy, and absolute true. We observed that all methods without GAT were surrounded by the corresponding methods with GAT in radar maps, suggesting that the methods with GAT outperform the methods without GAT in terms of precision, coverage, accuracy, and absolute true. These results demonstrate that the proposed graph node classification framework can enhance the identification of multi-functional peptides.

We also compared the performance of all competing methods on the MFBP (Figure 2C) and MFTP (Figure 2D) datasets. The pLM without GAT outperformed other features with or without GAT. And the performance of pLM was further improved by GAT, achieving the best performance with a precision of 0.777, coverage of 0.785, accuracy of 0.776, absolute true of 0.767, and absolute false of 0.082 on the MFBP dataset and a precision of 0.721, coverage of 0.722, accuracy of 0.679, absolute true of 0.605, and absolute false of 0.032 on the MFTP dataset. All results can be found in Tables S1 and S2. These results indicate that pLM can extract more comprehensive and high-quality features from peptide sequences than traditional features and other deep learning-based methods.

Thus, we constructed iMFP-LG by incorporating pLM as a feature extraction module and GAT as an identification module. To improve the generalization capability and avoid the over-fitting phenomenon, we also employed adversarial training to achieve better results in the final framework.

### iMFP-LG outperforms the state-of-the-art methods

We compared our proposed method, iMFP-LG, with several state-of-the-art methods, including four conventional machine learning-based methods (CLR [50], RAKEL [51], RBRL [52], and MLDF [53]) and four deep learning-based methods (MPMABP [38], MLBP [37], PrMFTP [39], and ETFC [40]). MPMABP and MLBP employed CNNs and RNNs for identifying multi-functional peptides. In addition to CNNs and RNNs, PrMFTP employed a multi-head self-attention module to further optimize the extracted features for predicting multi-functional therapeutic peptides. ETFC mitigated data imbalance using the focal dice loss function. The focal dice loss mitigates class imbalance by down-weighting the contribution of well-classified examples, allowing the model to focus more on hard-to-classify instances, which are often underrepresented. Note that due to the randomness of dividing the entire datasets into training and test datasets, we could not access their original training datasets. Besides, the compared methods didn’t provide the sensitivity and specificity analyses to each peptide category. Therefore, we reproduced the four deep learning-based methods in the same training dataset with our proposed method. All the reproduced results were comparable in performance to their reported results. For those methods that were not publicly available, we used only the results reported in the literature.

According to the performance comparison on the MFBP (Figure 2E) and MFTP (Figure 2F) datasets, iMFP-LG outperformed the state-of-the-art methods on both datasets in terms of all metrics, except for a comparable performance in terms of absolute false in the MFTP dataset. When compared on the MFBP dataset, iMFP-LG achieved a precision of 0.797, coverage of 0.803, accuracy of 0.796, absolute true of 0.788, and absolute false of 0.078, greatly outperforming the state-of-the-art method MLBP by 4.0%, 5.9%, 6.0%, 8.2%, and 1.5%, respectively. When compared on the MFTP dataset, iMFP-LG achieved a precision of 0.730, coverage of 0.730, accuracy of 0.689, and absolute true of 0.616, outperforming the state-of-the-art ETFC model by 0.9%, 1.3%, 0.8%, and 0.3%, respectively. All the methods achieved comparable performance in terms of absolute false. These results can be found in Tables S3 and S4. Overall, iMFP-LG comprehensively outperforms all multi-functional peptide prediction methods.

### iMFP-LG is more sensitive to peptide categories with small size and multi-functions

As there are 5 types of bioactive peptide functions in the MFBP dataset and 21 types of therapeutic peptide functions in the MFTP dataset, we further investigated the performance of competing methods in each peptide category.

**Figure 3:**
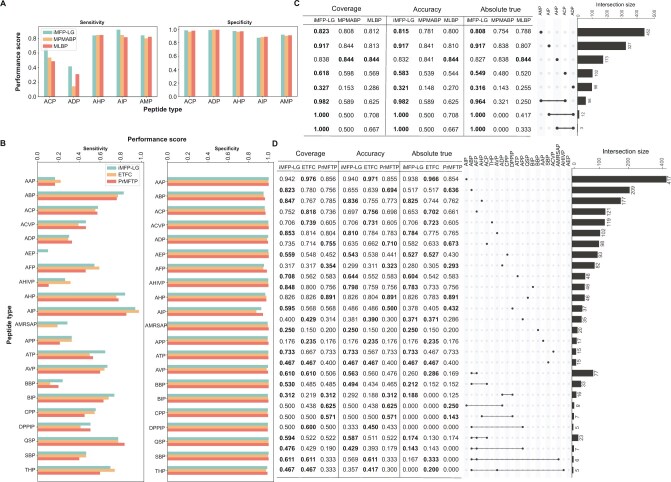
Performance comparison of different methods in each peptide category **A**. and **B**. Sensitivity and specificity of different competing methods on the MFBP (A) and MFTP (B) datasets. **C**. and **D**. Performance of different competing methods on mono-functional and multi-functional peptide prediction on the MFBP (C) and MFTP (D) datasets. The upset plot on the right shows the size of peptide categories in test datasets. For MFTP, we only show the peptide categories with size ≥ 5 and multi-functions ≤ 3. The table on the left shows the performance in terms of coverage, accuracy, and absolute true for corresponding peptide categories in the upset plot. The values in bold indicate the best performance across the compared methods. ACP, anti-cancer peptide; ADP, anti-diabetic peptide; AHP, anti-hypertensive peptide; AIP, anti-inflammatory peptide; AMP, anti-microbial peptide; AAP, anti-angiogenic peptide; ABP, anti-bacterial peptide; ACVP, anti-coronavirus peptide; AEP, anti-endotoxin peptide; AFP, anti-fungal peptide; AHIVP, anti-HIV peptide; AMRSAP, anti-MRSA peptide; APP, anti-parasitic peptide; ATP, anti-tubercular peptide; AVP, anti-viral peptide; BBP, blood-brain barrier peptide; CPP, cell-penetrating peptide; SBP, surface binding peptide; THP, tumor homing peptide.

We compared the sensitivity and specificity of iMFP-LG with other state-of-the-art methods on all kinds of peptide functions. The sensitivity and specificity were calculated by considering peptides with a function as positive samples and other peptides without that function as negative samples. The comparison results on the MFBP (**Figure 3**A) and MFTP (Figure 3B) datasets showed that all competing methods had high specificities on both datasets, indicating that these models have high specificities in single function classification. However, their sensitivities are low and differ largely. On the MFBP dataset in terms of sensitivity, iMFP-LG achieved the best results in the prediction of ACP, anti-diabetic peptide (ADP), anti-inflammatory peptide (AIP), and AMP. Specifically, it had a much higher sensitivity than the other two competing methods in two small categories ACP and ADP. On the MFTP dataset in terms of sensitivity, iMFP-LG also achieved better results than PrMFTP and ETFC across almost all peptide functions. Interestingly, PrMFTP failed to distinguish the peptides in three small categories anti-endotoxin peptide (AEP), anti-HIV peptide (AHIVP), and anti-MRSA peptide (AMRSAP), and ETFC failed to predict the AEP. Nonetheless, iMFP-LG greatly improved the prediction performance. These results demonstrate that iMFP-LG is more sensitive to peptide categories of small size by taking advantage of pLM to learn high-quality peptide representations.

We then evaluated the performance of iMFP-LG in identifying both mono-functional and multi-functional peptides on the MFBP (Figure 3C) and MFTP (Figure 3D) datasets. On the MFBP dataset, iMFP-LG outperformed the competing methods across all peptide categories except for AHP. Notably, for multi-functional peptides, iMFP-LG achieved an absolute true of 1.00 in both ADP_AIP and ADP_AHP functions, and an absolute true of 0.964 in the ACP_AMP functions. On the MFTP dataset, iMFP-LG also achieved better performance than the competing methods in most peptide categories, especially in the multi-functional peptides. These results demonstrate that iMFP-LG is more sensitive to peptide categories with multi-functions by taking advantage of GAT to capture the complex functional relationships.

### Model ablation study

We then explored the contribution of each module to our proposed method using ablation experiments on two datasets. There are three important modules in iMFP-LG: adversarial training, GAT, and pre-trained pLM. We built several variants of iMFP-LG with and without these modules.

**Tables 1** and **2** show the performance of iMFP-LG and its variants on the MFBP and MFTP datasets. All variants were consistent with the experimental settings except for the learning rate in the ablation study. Because pLMs with random initialization are difficult to train at small learning rates, we set the learning rate to 1E–5 in the “w/o pretrain” model and 5E–5 for all others. As can be seen, removing any module from the proposed model reduces its performance. On the MFBP dataset, the performance of the “w/o pretrain” model was the most deteriorated with an accuracy of 0.752 and absolute true of 0.733. The “w/o ad” model showed a reduction in performance after removing the adversarial training with an accuracy of 0.776 and absolute true of 0.767. Similarly, the performance of the “w/o GAT” model was dropped with an accuracy of 0.784 and absolute true of 0.776. On the MFTP dataset, the performance of the “w/o pretrain” model decreased drastically, and its accuracy and absolute true decreased by 7.1% and 6.9%, respectively. The performance of both “w/o GAT” and “w/o ad” models also decreased obviously in terms of all metrics. Ablation studies reveal that all three modules have critical contributions to improving the performance of multi-functional peptide prediction, especially the GAT and pLM modules.

**Table 1:** The performance of iMFP-LG and its variants on the MFBP dataset

Model	Precision ↑	Coverage ↑	Accuracy ↑	Absolute true ↑	Absolute false ↓
iMFP-LG	**0.797**	**0.803**	**0.796**	**0.788**	**0.078**
w/o ad^a^	0.777	0.785	0.776	0.767	0.082
w/o GAT^b^	0.785	0.791	0.784	0.776	0.080
w/o pretrain^c^	0.754	0.769	0.752	0.733	0.095

*Note*: The highest values are highlighted in bold. **↑** means that a larger value is better on this metric; **↓** means that a smaller value is better on this metric; ^a^ w/o ad is a variant in which the adversarial training is not used during training process; ^b^ w/o GAT is a variant without GAT; ^c^ w/o pretrain is a variant in which the protein language model is re-initialized randomly instead of using pre-trained weights. GAT, graph attention network; MFBP, multi-functional bioactive peptide.

**Table 2:** The performance of iMFP-LG and its variants on the MFTP dataset

Model	Precision ↑	Coverage ↑	Accuracy ↑	Absolute true ↑	Absolute false ↓
**iMFP-LG**	**0.730**	**0.730**	**0.689**	**0.616**	**0.032**
w/o ad^a^	0.721	0.722	0.679	0.605	0.032
w/o GAT^b^	0.709	0.705	0.667	0.598	0.033
w/o pretrain^c^	0.658	0.657	0.618	0.547	0.036

*Note*: The highest values are highlighted in bold. **↑** means that a larger value is better on this metric; **↓** means that a smaller value is better on this metric; ^a^ w/o ad is a variant in which the adversarial training is not used during training process; ^b^ w/o GAT is a variant without GAT; ^c^ w/o pretrain is a variant in which the protein language model is re-initialized randomly instead of using pre-trained weights. MFTP, multi-functional therapeutic peptide.

### Interpretability of iMFP-LG

The intuition behind iMFP-LG is to extract key sequence patterns using pLM and capture intricate multi-functional relationships via GAT. Thus, we can unveil its decision process of how to assign multi-functional labels to peptides by visualizing the distribution of peptide representations, sequence motifs, and multi-function relationships.

**Figure 4:**
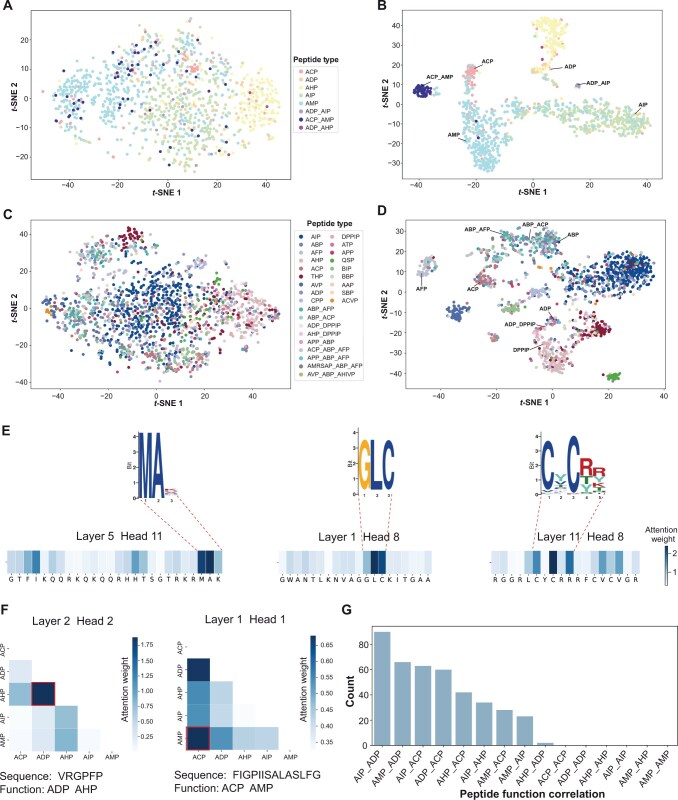
Illustration of the interpretability of iMFP-LG *t*-SNE visualization of the distribution of peptide representations obtained by pre-trained and fine-tuned pLMs on the MFBP dataset (**A** and **B**) and the MFTP dataset (**C** and **D**), respectively. **E**. Three AMP cases where the sequence patterns captured by pLM are matched with the motifs discovered by STREME. **F**. Two multi-functional peptide cases in which the learned graph node relationships are consistent with their true function labels. **G**. Peptide function correlation between the maximum values within each GAT attention matrix. *t*-SNE, *t*-distributed Stochastic Neighborhood Embedding.

All peptide representations were extracted by the pLM from the MFBP and MFTP test datasets and visualized using *t*-distributed Stochastic Neighborhood Embedding (*t*-SNE) [54]. We compared the distribution of peptide representations extracted by the pre-trained and fine-tuned pLMs from the MFBP and MFTP datasets, respectively (**Figure 4**A–D). Although the pre-trained model initially identified different multi-functional peptides, the fine-tuned model exhibited more distinct ability to cluster peptides with the same functions into categories. Interestingly, the peptide clusters with multiple functions lay between corresponding clusters of mono-functional peptides. For example, in the distribution of multi-functional bioactive peptides (Figure 4B), the peptide cluster with ADP_AIP function lay between the peptide cluster with ADP function and the peptide cluster with AIP function. And the peptide cluster with ACP_AMP function was also close to the cluster with ACP function and the cluster with AMP function. A similar phenomenon was also observed in the distribution of multi-functional therapeutic peptides (Figure 4D), such as the distribution of the peptide clusters with ABP, ACP, and ABP_ACP functions as well as the peptide clusters with ADP, dipeptidyl peptidase IV peptide (DPPIP), and ADP_DPPIP functions. These results suggest that iMFP-LG has the capability to map peptides into a robust representation space, demonstrating spatial interpretability with the continuity property: two similar multi-functional peptides should not be projected as two distant points in the representation space. This advancement provides an opportunity to explore the phylogenetic relationships of multi-functional peptides in relation to mono-functional peptides.

To extract sequence patterns from the attention mechanism, we calculated the importance of each amino acid in a peptide sequence. Each sequence is transformed into an embedding by 12 attention heads in each of 12 layers, resulting in a total of 144 attention heads in the pLM. The weight $\beta_{i,j}$ in an attention head matrix indicates how much information from the amino acid in position j should be used when computing the representation of amino acid in position i. We summed the attention of all amino acids to amino acid j as its effect $d_{j}=\sum_{i=1}^{n}\beta_{i,j}$ to the entire peptide sequence. A higher $d_{j}$ value suggests that amino acid j is more important in the peptide sequence. Figure 4E shows three AMP cases where the sequence patterns captured by attention mechanism were matched with the motifs discovered by STREME. The parameters of STREME are shown in Table S7. We also used the attention visualization tool bertviz [55] to reveal the detail of the attention pattern (Figure S1). These results suggest that iMFP-LG can identify functional regions of peptides.

To interpret the intricate correlations captured by GAT, we visualized the node connections across two layers and six heads. The node connection $r_{i,j}=\gamma_{i,j}+\gamma_{j,i}$ is calculated from the graph attention matrix γ, where each element $\gamma_{i,j}$ describes the importance of edge from node i to node j. Figure 4F shows two multi-functional peptide cases, where the node connections with the highest values matched with their true function labels. To find more reliable functional correlations in GAT, we also calculated the pairwise correlations of peptide functions by counting the occurrences of two function labels that have the maximum node connection values in all graph attention matrices. We assessed the captured node correlations of AIP_ADP training samples (Figure 4G). The functions AIP and ADP have the highest counts, indicating that they have the strongest correlation. These results indicate that iMFP-LG can learn the correlations among different peptide function labels.

### Discovery of novel multi-functional peptides

Regarding the outstanding performance of iMFP-LG in identifying multi-functional peptides, especially on the MFBP dataset where it achieves an absolute true of 0.964 in ACP_AMP function, we employed iMFP-LG to screen novel candidate peptides with both ACP and AMP functions from millions of known peptides in the UniRef90 database.

**Figure 5:**
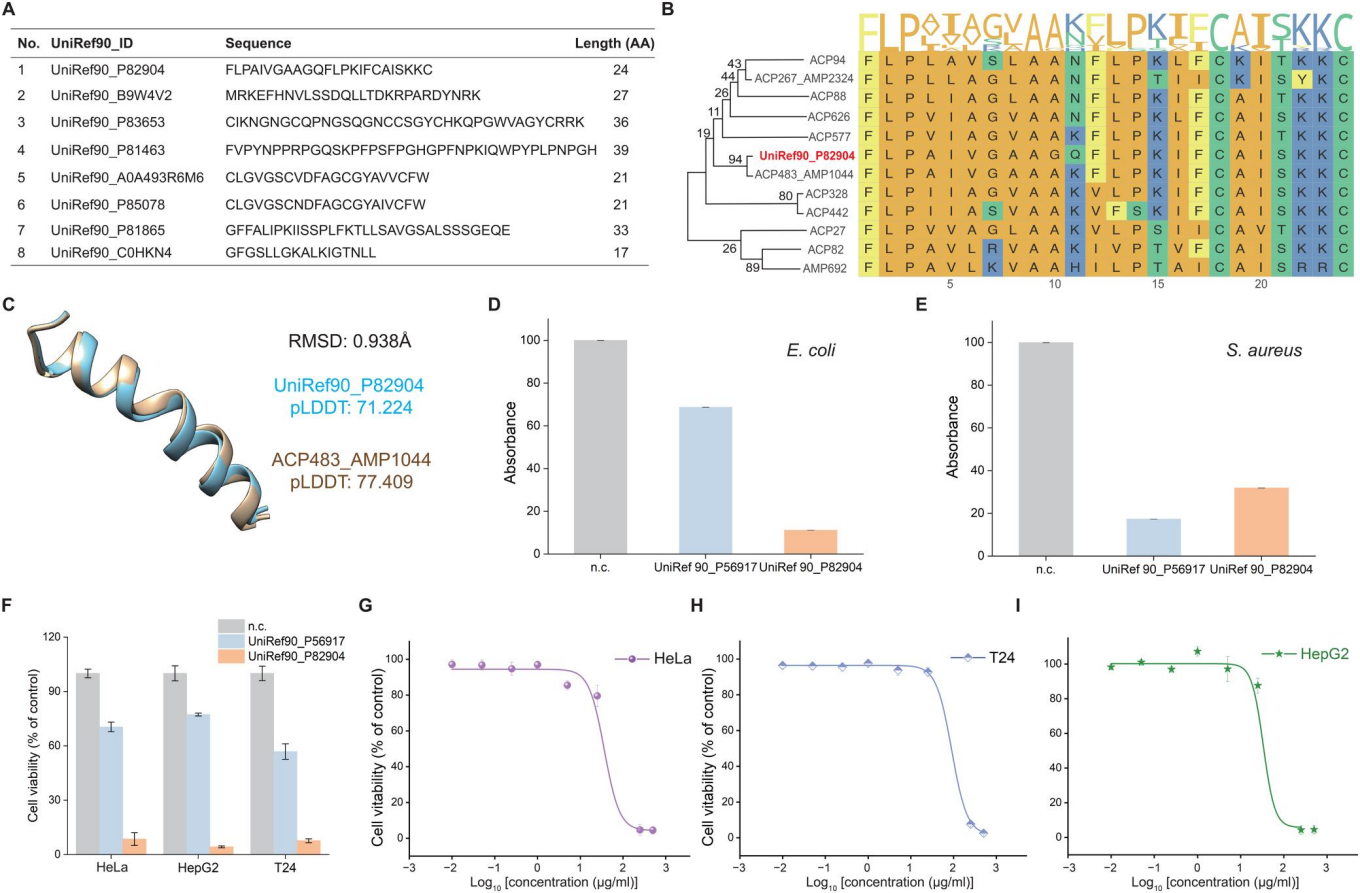
Multi-functional peptides discovered by iMFP-LG from UniRef90 **A**. Candidate peptides with both anti-cancer and anti-microbial functions screened from UniRef90 by iMFP-LG. **B**. Multiple sequence alignment and phylogenetic tree of the candidate peptide UniRef90_P82904. **C**. Structure alignment of the candidate peptide UniRef90_P82904 with its homologous sequence. **D**. and **E**. Bacterial inhibition effects of peptides UniRef90_P56917 and UniRef90_P82904 on *E. coli* (D) and *S. aureus* (E). **F**. Cytotoxic effects of peptides UniRef90_P56917 and UniRef90_P82904 on HeLa, HepG2, and T24 tumor cells. **G**.–**I**. Dose-dependent cytotoxic effects of the candidate peptide UniRef90_P82904 on HeLa (G), T24 (H), and HepG2 (I) tumor cells. AA, amino acid; n.c., negative control.

Considering that UniRef90 contains 166,459,614 protein sequences, we filtered out any sequences longer than 40 amino acids or short than 4 amino acids to specifically focus on peptides. This resulted in a dataset of 1,077,593 peptide sequences, which were then fed into iMFP-LG. To discover novel multi-functional peptides, we re-trained iMFP-LG on the entire MFBP dataset with the same hyperparameters. To obtain candidate peptides with high confidence, the classification threshold was set to 0.95 for both ACP and AMP functions. After removing duplicated peptides that appeared in the MFBP dataset, we ultimately achieved 8 candidate peptides (**Figure 5**A). The functions of these peptides were further verified by searching their homologous sequences (Figure 5B, Figure S2A) through multiple sequence alignment [56], using candidate sequences as queries and peptides with ACP or AMP function in the MFBP dataset as targets. For the candidates that had ≥ 4 homologous sequences, we successfully constructed their phylogenetic trees, demonstrating that these candidates have an evolutionary relationship with known ACPs and AMPs. We then predicted their structures using ESM-Fold [57], and performed a structural alignment with their homologous sequences. The peptide UniRef90_P82904 exhibited exceptional alignment results (Figure 5C), while the peptides UniRef90_P83653 and UniRef90_B9W4V2 had no similar structures to their homologous sequences (Figure S2B and C).

To further assess the functions of these three candidate peptides, we subsequently conducted biological experiments. A positive control (UniRef90_P56917) with both AMP and ACP functions [58] was randomly selected. Peptides at a concentration of 500 μM were added to the cell culture for 24 h, followed by the 3-(4,5-dimethylthiazol-2-yl)-2,5-diphenyltetrazolium bromide (MTT) cytotoxicity assay. Three independent replicates were conducted. The results showed that the peptide UniRef90_P82904 had an excellent anti-bacterial effect against both *Escherichia coli* and *Staphylococcus aureus* (Figure 5D and E). The other two peptides, UniRef90_P83653 and UniRef90_B9W4V2, exhibited no anti-bacterial activity against either *E. coli* or *S. aureus* (Figure S2D and E). To assess the anti-cancer activity of these peptides, cell viability tests were performed on three kinds of human tumor cell lines, including bladder cancer (T24), cervical cancer (HeLa), and liver cancer (HepG2). We found that the peptide UniRef90_P82904 had the strongest anti-cancer activity against all three tested cell lines (Figure 5F), and the peptide UniRef90_P83653 demonstrated strong anti-cancer activity against HeLa cells but had weak anti-cancer activity against T24 and HepG2 cells (Figure S2F). The peptide UniRef90_B9W4V2 exclusively exhibited weak anti-cancer activity against T24 cells, and had no effect on the other two tested cell lines (Figure S2F). Subsequently, the peptide UniRef90_P82904 was chosen for a dose-dependence analysis in a 24-h assay to evaluate its cytotoxic effect against three tumor cell lines using the standard MTT assay. As shown in Figure 5G–I, the cell viability decreased to as low as 4.7%, 7.7%, and 4.3% at 250 μg/ml against HeLa, T24, and HepG2 cancer cells, demonstrating the promising capability for ablation of these three cancer cells. The outcomes of biological experiments are consistent with the computational screening results of iMFP-LG, indicating that iMFP-LG has a strong potential to discover novel multi-functional peptides.

## Conclusion

In this study, we proposed a method iMFP-LG for discovering multi-functional peptides. iMFP-LG converts multi-label predictions to graph node classifications based on pLM and GAT. Comparison results on the MFBP and MFTP datasets showed that iMFP-LG outperformed the state-of-the-art methods, especially for small and multi-functional peptide categories. iMFP-LG is also interpretable by visualizing the patterns captured by the attention mechanisms of pLM and GAT. Subsequently, a peptide discovery pipeline was established based on iMFP-LG to screen for novel multi-functional peptides. Eight candidate peptides with both anti-microbial and anti-cancer functions were discovered from the UniRef90 database. Further biological experiments demonstrated the promising anti-cancer and anti-bacterial activities of the candidate peptides, indicating that iMFP-LG has a strong potential to discover novel multi-functional peptides.

In future studies, we plan to integrate function-related features, structure information, and physic-chemical properties to strengthen the capability of graph nodes. Graph networks can be developed to delve further into the connections between various functional features [10]. Although deep learning-based predictions have achieved significant success, experimental validation of the predicted functions of peptides remains necessary for future study. In our opinion, other multi-label bioinformatics tasks [59] can effectively use our technique as an extension.

## Materials and methods

### Datasets

We evaluated the performance of our proposed method on two widely used multi-functional peptide datasets, MFBP [37] and MFTP [39]. Both were collected from the literature by searching specific keywords in Google Scholar. Notably, all peptide functions are based on the experimental results available to date. It is highly possible that some peptides have potential functions that have not yet been unveiled. We randomly sampled 80% of the data as training data, and the remaining 20% was the test data.

The MFBP dataset was collected by searching the keyword ‘bioactive peptides’ in Google Scholar in June 2020. It contains 5986 bioactive peptides with 5 different functional attributes, including ACP, ADP, AHP, AIP, and AMP. For each functional peptide category, CD-HIT [60] was applied to remove sequences with similarity greater than 90% in order to avoid redundancy and homology bias. The majority of bioactive peptides only have one type of activity, a few peptides have two types of activity jointly, and no peptide has more than two types of activities at the same time. The distribution of peptide categories is shown in Figure S3.

The MFTP dataset was collected by searching the keyword ‘therapeutic peptides’ in Google Scholar in July 2021. The collected data were preprocessed using following criteria: (1) sequences containing non-standard amino acids were discard; (2) peptides longer than 50 amino acids or shorter than 5 amino acids were removed [61], since more than 97% of functional peptides are shorter than 50 amino acids according to the distribution of sequence lengths of AMPs in the APD3 dataset [62]; and (3) peptides with fewer than 40 samples were deleted. After data processing, there were 9874 therapeutic peptides with 21 different functional attributes, including anti-angiogenic peptide (AAP), ABP, ACP, anti-coronavirus peptide (ACVP), ADP, AEP, anti-fungal peptide (AFP), AHIVP, AHP, AIP, AMRSAP, anti-parasitic peptide (APP), anti-tubercular peptide (ATP), anti-viral peptide (AVP), blood-brain barrier peptide (BBP), biofilm-inhibitory peptide (BIP), cell-penetrating peptide (CPP), DPPIP, quorum-sensing peptide (QSP), surface binding peptide (SBP), and tumor homing peptide (THP). The distribution of peptides for each type is shown in Figure S4.

### Peptide representation module

Here, the pre-trained pLM TAPE [49] was employed to generate peptide representations. TAPE is a bidirectional encoder representation from a transformer-based language model [31] constructed with a 12-layer Transformer encoder and 12 attention heads in each layer. The core of attention mechanism can be formulated as Equations 1 and 2:

(#1) Q=XWQ, K=XWK, V=XWV

 

(#2) AttentionQ, K, V=softmaxQKTdkV

where $X\in R^{L\times d_{m}}$ is an embedding of a peptide sequence. The sequence embedding X is transformed to a query matrix $Q\in R^{L\times d_{k}}$, a key matrix $K\in R^{L\times d_{k}}$, and a value matrix $V\in R^{L\times d_{k}}$ by linear transformation, where $W^{Q},\;W^{K},\;W^{V}\in R^{d_{m}\times d_{k}}$ are the learnable parameters of the attention layer. The attention scores are the normalized dot product of the key and query vectors in Equation 2. The multi-head attention is an extension of the attention mechanism to catch rich information from multiple projections, and it can be formulated as Equations 3 and 4:

(#3) headi=AttentionXWiQ,XWiK,XWiV

 

(#4) MultiheadAttentionQ,K,V=Concatheadi,…,headhWO

where $W_{i}^{Q},W_{i}^{K},W_{i}^{V}\in R^{d_{m}\times d_{k}}$ are the learnable parameters in $\mathrm{hea}d_{i}$. The output of the multi-head attention layer is obtained by concatenating the outputs of all attention heads, followed by a linear transformation using $W^{O}\in R^{{hd}_{v}\times d_{m}}$.

TAPE was pre-trained by masked-token prediction on the Pfam [63] corpus, which contains more than 31 million protein domains. The aim of masked-token prediction is to predict randomly masked amino acids based on other amino acids in the protein. Thus, TAPE can model the general relationships between amino acid residues. In this study, the parameters in TAPE were trainable and updated during the training process of the whole framework. Subsequently, the pLM was fine-tuned to identify multi-functional peptides in the training datasets. We used the output of the pooler layer in TAPE as peptide representations with dimension of 768.

### Graph classification module

The graph classification module is used to transform the multi-label classification problem into a graph node classification problem. It consists of three parts: a node feature encoder, a GAT, and a node classifier.

The node feature encoder constructs a linear transform layer for each graph node to convert the peptide representation to the corresponding node representation, which can be formulated as Equation 5:

(#5) hi=dropoutWi·p

where $h_{i}\in R^{d_{\mathrm{pLM}}}$represents the feature of node i, $p\in R^{d_{\mathrm{pLM}}}$ is the peptide representation, and $W_{i}\in R^{d_{\mathrm{pLM}}\times d_{\mathrm{pLM}}}$is the trainable parameter of the linear transformation layer. Specifically, a peptide representation with the dimension of 768 was converted into 5 node representations for the MFBP dataset and 21 node representations for the MFTP dataset. All node representations are dimension of 768.

The GAT is a multi-head attention-based graph neural network for fine-tuning node representation by learning relationships among peptide functions. The graph nodes represent the different peptide functions and the edges represent the associations between two functions. The node representation is updated by the GAT as Equation 6:

(#6) hi′=∑j∈NiαijWhj

where $h_{i}^{\prime }$ represents the updated node representation, $W\in R^{d\times d_{\mathrm{pLM}}}$ is the transformation matrix, α represents the attention matrix, and$\;\alpha_{ij}$ indicates the importance of the node j to node i. It can be calculated as Equation 7:

(#7) αij= exp(LeakyReLU(αT[Whi║║Whj]))∑k∈Ni exp LeakyReLU(αT[Whi║║Whk])

where $\alpha^{T}\in R^{2d}$ is the parameter matrix of the attention mechanism, and ║║ denotes the concatenation operation. Multi-head attention [64] is used in GAT. The final node features are obtained by concatenating K independent attention heads, which can be formulated as Equation 8:

(#8) hi′=║║k=1Kσ∑j∈NiαijkWkhj

where K is the number of attention heads, σ represents the activation functions, ║║ denotes the concatenation operation, $\alpha_{ij}^{k}\;$and $W^{k}$ are attention coefficients and weight matrix in k-th attention mechanism. In this study, we created 2-layer fully connected graphs among function labels, with all edge weights initialized to 1 and each layer having 6 attention heads.

The node classifier is a set of binary predictors used for the final prediction of function labels. The updated node representations from GAT are fed into the corresponding binary predictors to predict whether the peptide has the corresponding function or not. In this study, each binary predictor has a hidden layer and an output layer with sigmoid activation function, which can be formulated as Equation 9:

(#9) resi=sigmoidWi⋅hi′

where $W_{i}\in R^{1\times d_{\mathrm{pLM}}}$ is learnable in the hidden layer. In practice, we concatenated the outcomes of each classification to calculate the loss using the binary cross-entropy loss function.

### Adversarial training

In order to improve the protein language representations’ capability and avoid the overfitting phenomenon, we employed an adversarial training strategy called Fast Gradient Method (FGM) [65,66] during the training process.

FGM introduces an adversarial perturbation to the embeddings of amino acids according to the updated gradients. The adversarial perturbation $r_{\mathrm{adv}}$ can be defined as Equations 10 and 11.

(#10) radv=-εg/║║g║║2

 

(#11) $$\begin{array}{c} g={▽}_{e}\log p\left(y|e;\hat{\theta }\right) \end{array}$$

where e is the embedding of peptide sequences; y represents true function labels; $p\left(y|e;\theta \right)$ represents the conditional probability of y given e; *θ* represents the parameter of the model, and $\hat{\theta }$ is a constant set to the current parameters of the model; and ε represents the shared norm constraint of adversarial loss.

The goal of adversarial training is to minimize the original loss without perturbation and the adversarial loss with the adversarial perturbation (Figure 1E). The procedure of these two goals can be formulated as Equations 12 and 13, respectively.

(#12) $$\begin{array}{c} L_{\mathrm{init}}\left(\theta \right)=-\frac{1}{N}\sum_{i=1}^{N}y_{i}\log p\left(y_{i}|x_{i};\theta \right) \end{array}$$

 

(#13) $$\begin{array}{c} L_{\mathrm{adv}}\left(\theta \right)=-\frac{1}{N}\sum_{i=1}^{N}y_{i}\log p\left(y_{i}|x_{i}+r_{\mathrm{adv},i};\theta \right) \end{array}$$

where N is the number of batch size, $x_{i},\;y_{i},\;\mathrm{and}\;r_{\mathrm{adv},i}$ are the input peptide sequence, label, and perturbation on the i-th sample in a batch, respectively, and θ denotes the model’s parameters.

### Evaluation metrics

In order to make a full and fair comparison with the state-of-the-art methods, we adopt five evaluation metrics, including precision, coverage, accuracy, absolute true, and absolute false. These metrics are defined as Equations 14–18:

(#14) Precision=1N∑i=1N║║Li∩Li*║║║║Li*║║

 

(#15) Coverage=1N∑i=1N║║Li∩Li*║║║║Li║║

 

(#16) Accuracy=1N∑i=1N║║Li∩Li*║║║║Li∪Li*║║

 

(#17) Absolute true=1N∑i=1N▵Li,Li*

 

(#18) Absolute false=1N∑i=1N║║Li∪Li*║║-║║Li∩Li*║║M

where N represents the total number of peptide sequences in the dataset, M denotes the number of labels, ∩ and ∪ are the intersect and union operations in the set theory, ║║S║║ represents the size of a set *S*, $L_{i}$ denotes the true label subset of the i-th peptide sample, $L_{i}^{*}$ represents the predict label subset of i-th sample by the classifier, and $▵\left(L_{i},L_{i}^{*}\right)$ can be formulated as Equation 19:

(#19) ▵Li,Li*=0, if Li is identical to Li* 1, other

We also employed the sensitivity and specificity metrics [37,67] to further compare the performance on each peptide function, which can be formulated as Equations 20 and 21:

(#20) $$\begin{array}{c} Sensitivity=\frac{TP}{TP+FN} \end{array}$$

 

(#21) $$\begin{array}{c} Specificity=\frac{TN}{TN+FP} \end{array}$$

where the numbers of true positives, true negatives, false positives, and false negatives are denoted by TP, TN, FP, and FN, respectively. When calculating the sensitivity and specificity of a specific functional peptide, that class of peptide is considered a positive sample and other peptides without that function are considered negative samples.

### Implementation details

Our proposed model was implemented using PyTorch1.12 in a computing server equipped with an Intel(R) Xeon(R) Gold 6248R CPU @ 3.00 GHz and an NVIDIA A100 GPU. The proposed model was trained in 100 epochs with batch size 32 and AdamW optimizer [68]. Since the pLM was already pre-trained, we set a small learning rate of 5E–5 to fine-tune it. The learning rates of the GAT were 1E–3 and 5E–4 for the MFBP and MFTP datasets respectively. We constructed fully-connected graphs with 5 and 21 nodes for the MFBP and MFTP datasets, respectively, and both initialized with edge weights of 1. The number of attention heads in GAT was 6 and the dimension of node features in each attention head was 128. The final node features in GAT had a dimension of 768, the same as the representation obtained from the language model. The norm constraint ε of adversarial training was set to 0.5. In order to reduce the effects caused by the random initialization of the deep learning framework and to maintain a consistent setting with the compared methods [37,39], all models were trained 10 times repeatedly, and the prediction results were averaged as the final prediction for testing samples.

### Peptide synthesis and biological experiments

#### Peptide synthesis

The peptides used in this study were synthesized via solid-phase peptide synthesis (Beijing Liuhe Bada Gene Technology, China), and their precise molecular weights were determined using mass spectrometry. The purity of all peptides was assessed by high-performance liquid chromatography, and all samples showing a purity greater than 90%.

#### Bacterial inhibition experiment

An *S. aureus* strain was streaked on Luriae-Bertani (LB) agar medium and incubated at 37°C overnight. An individual colony was picked into LB culture medium and shaken at 120 r/min at 37°C overnight. The LB bacterial suspension was diluted to the predetermined starting concentration [optical density at 600 nm (OD600) = 0.1] and then further diluted 1000 times for the inhibition test. Freeze-dried peptide powder was thawed and dissolved in double-distilled water to 50 mM. Three experimental groups were set up to test peptide anti-bacterial activity: (1) blank control group, 50 μl of LB solution; (2) bacterial control group, 25 μl of LB solution and 25 μl of bacterial solution; and (3) peptide group, 23 μl of LB solution, 25 μl of bacterial solution, and 2 μl of peptide solution (500 µM). All experiments were performed on 96-well plates with each single well containing 50 μl of final volume. After culture at 37°C for 12 h, the absorbance value of each well was determined by using a microplate reader at OD600. All experiments were performed with three independent replicates.

#### Tumor inhibition experiment

Tumor inhibition effects of peptides were determined using MTT cytotoxicity assay for T24, HeLa, and HepG2 cell lines. Exponentially growing cells were seeded in 96-well microtiter plates at a density of approximately $5\times 10^{3}$ cells per well. After a 24-h incubation at 37°C with 5% CO_2_, the medium was replaced with fresh medium, and peptides at final concentration of 500 μg/ml each were added, followed by 24-h incubation. Cell viability was monitored by the addition of 5 mg/ml MTT solution, and the absorbance value was measured at OD570 after incubation for 4 h. Each peptide experiment was performed with three replicates. The optical density of wells containing cells cultured without peptides was assumed to represent 100% cell viability.

## Code availability

The associated code is available at GitHub (https://github.com/chen-bioinfo/iMFP-LG). The code has also been submitted to BioCode at the National Genomics Data Center, Beijing Institute of Genomics, Chinese Academy of Sciences / China National Center for Bioinformation (BioCode: BT007494), which is publicly accessible at https://ngdc.cncb.ac.cn/biocode/tools/BT007494.

## Supplementary Material

qzae084_Supplementary_Data

## CRediT author statement

**Jiawei Luo:** Conceptualization, Data curation, Formal analysis, Investigation, Methodology, Project administration, Resources, Software, Visualization, Writing – original draft. **Kejuan Zhao:** Resources, Visualization, Formal analysis, Writing – original draft. **Junjie Chen:** Conceptualization, Formal analysis, Methodology, Funding acquisition, Supervision, Writing – original draft, Writing – review & editing. **Caihua Yang:** Methodology, Resources. **Fuchuan Qu:** Formal analysis. **Yumeng Liu:** Writing – review & editing. **Xiaopeng Jin:** Writing – review & editing. **Ke Yan:** Writing – review & editing. **Yang Zhang:** Supervision, Writing – review & editing. **Bin Liu:** Writing – review & editing. All authors have read and approved the final manuscript.
