# Supplementary material for: iMFP-LG: Identify Novel Multi-functional Peptides Using Protein Language Models and Graph-based Deep Learning
Source: Genomics Proteomics Bioinformatics. 2024 Nov 25;22(6):qzae084. doi: 10.1093/gpbjnl/qzae084 (PMC12011362; doi:10.1093/gpbjnl/qzae084)
Supplement: qzae084_Supplementary_Data [file qzae084_supplementary_data.zip › Table S4.docx]

**Table S4 The performance comparison of our proposed method iMFP-LG with the state-of-the-art methods on the MFTP dataset**

| **Model** | **Precision ↑** | **Coverage ↑** | **Accuracy ↑** | **Absolute true ↑** | **Absolute false ↓** |
| --- | --- | --- | --- | --- | --- |
| BR | 0.427 | 0.437 | 0.394 | 0.325 | 0.05 |
| CLR | 0.418 | 0.428 | 0.387 | 0.32 | 0.047 |
| RAKEL | 0.349 | 0.317 | 0.307 | 0.265 | 0.052 |
| RBRL | 0.513 | 0.507 | 0.478 | 0.426 | 0.057 |
| PrMFTP | 0.699 | 0.669 | 0.651 | 0.593 | 0.031 |
| ETFC | 0.721 | 0.717 | 0.681 | 0.613 | 0.036 |
| iMFP-LG | 0.730 | 0.730 | 0.689 | 0.616 | 0.032 |

*Note*: **↑** means a larger value is better on this metric; **↓** means a smaller value is better on this metric.
