# Supplementary material for: iMFP-LG: Identify Novel Multi-functional Peptides Using Protein Language Models and Graph-based Deep Learning
Source: Genomics Proteomics Bioinformatics. 2024 Nov 25;22(6):qzae084. doi: 10.1093/gpbjnl/qzae084 (PMC12011362; doi:10.1093/gpbjnl/qzae084)
Supplement: qzae084_Supplementary_Data [file qzae084_supplementary_data.zip › supplementary material captions.docx]

**Supplementary material**

**Figure S1 Visualization of attention weights**

The 3 AMP cases to show attention weights between amino acids in a peptide sequences visualized via bertviz. The darker colors represent higher attention scores.

**Figure S2 Discovery of novel multi-functional peptides by using iMFP-LG from UniRef90**

**A.** MSA and polygenetic trees of candidate peptides. **B.** and **C.** Structure alignment of homologous sequences with the closest evolutionary distance in polygenetic tree. **D.** and **E.** Bacterial inhibition effect of peptides UniRef90_P56917, UniRef90_P83653 and UniRef90_B9W4V2 on E. coli and S. aureus bacteria strains. **F.** Cytotoxic effect of peptides UniRef90_P56917, UniRef90_P83653 and UniRef90_B9W4V2 on T24, Hela, HepG2 cells. Peptides at 500 $\text{μg/}\mathrm{ml}$ were added to cell culture for 24 hours, followed by MTT cytotoxicity assay. The results are the average of three independent replicates.

**Figure S3 The distribution of multi-functional bioactive peptides in MFBP dataset**

The vertical bar on the upper side shows the size of mono-functional and multi-functional peptide categories. The functions of each category are marked with the dots. The bottom horizontal bar shows the size of peptides in each function set.

**Figure S4 The distribution of multi-functional therapeutic peptides in MFTP dataset**

The horizontal bar on the left side shows the size of mono-functional and multi-functional peptide categories. The functions of each category are marked with the dots. The vertical bar shows the size of peptides in each function set. (zoom in to see the details)

**Tables**

**Table S1 The performance comparison of different feature extraction methods with and without GAT on MFBP dataset**

**Table S2 The performance comparison of different feature extraction methods with and without GAT on MFTP dataset**

**Table S3 The performance comparison of our proposed method iMFP-LG with the state-of-the-art methods on the MFBP dataset**

**Table S4 The performance comparison of our proposed method iMFP-LG with the state-of-the-art methods on the MFTP dataset**

**Table S5 The performance of the MFBP experiment model with 10 repetitions on MFBP test set**

**Table S6 The performance of the MFTP experiment model with 10 repetitions on MFTP test set**

**Table S7 The settings of STREME for finding the motif of AMP in MFBP dataset**
