# Supplementary material for: iMFP-LG: Identify Novel Multi-functional Peptides Using Protein Language Models and Graph-based Deep Learning
Source: Genomics Proteomics Bioinformatics. 2024 Nov 25;22(6):qzae084. doi: 10.1093/gpbjnl/qzae084 (PMC12011362; doi:10.1093/gpbjnl/qzae084)
Supplement: qzae084_Supplementary_Data [file qzae084_supplementary_data.zip › Table S6.docx]

**Table S6 The performance of the MFTP experiment model with 10 repetitions on MFTP test set**

| **Model** | **Precision ↑** | **Coverage ↑** | **Accuracy ↑** | **Absolute true ↑** | **Absolute false ↓** |
| --- | --- | --- | --- | --- | --- |
| Model0 | 0.695 | 0.713 | 0.657 | 0.575 | 0.039 |
| Model1 | 0.695 | 0.712 | 0.657 | 0.575 | 0.038 |
| Model2 | 0.693 | 0.713 | 0.660 | 0.582 | 0.038 |
| Model3 | 0.688 | 0.701 | 0.652 | 0.575 | 0.037 |
| Model4 | 0.698 | 0.716 | 0.660 | 0.575 | 0.039 |
| Model5 | 0.695 | 0.713 | 0.658 | 0.574 | 0.039 |
| Model6 | 0.711 | 0.728 | 0.674 | 0.593 | 0.037 |
| Model7 | 0.707 | 0.713 | 0.662 | 0.582 | 0.037 |
| Model8 | 0.702 | 0.717 | 0.665 | 0.588 | 0.037 |
| Model9 | 0.700 | 0.705 | 0.658 | 0.581 | 0.038 |
| Model_avg_ | 0.730 | 0.730 | 0.689 | 0.616 | 0.032 |

*Note*: ↑ means a larger value is better on this metric; ↓ means a smaller value is better on this metric. Model0-9 mean the results of the model repeated 10 times with different random seeds. Model_avg_ means averaged the results of 10 model (Model0-9) predictions as the final prediction for testing samples.
