# Supplementary material for: iMFP-LG: Identify Novel Multi-functional Peptides Using Protein Language Models and Graph-based Deep Learning
Source: Genomics Proteomics Bioinformatics. 2024 Nov 25;22(6):qzae084. doi: 10.1093/gpbjnl/qzae084 (PMC12011362; doi:10.1093/gpbjnl/qzae084)
Supplement: qzae084_Supplementary_Data [file qzae084_supplementary_data.zip › Table S3.docx]

**Table S3 The performance comparison of our proposed method iMFP-LG with the state-of-the-art methods on the MFBP dataset**

| **Model** | **Precision ↑** | **Coverage ↑** | **Accuracy ↑** | **Absolute true ↑** | **Absolute false ↓** |
| --- | --- | --- | --- | --- | --- |
| CLR | 0.667 | 0.677 | 0.666 | 0.655 | 0.133 |
| RAKEL | 0.649 | 0.648 | 0.648 | 0.647 | 0.141 |
| MLDF | 0.649 | 0.649 | 0.648 | 0.646 | 0.119 |
| RBRL | 0.650 | 0.651 | 0.649 | 0.646 | 0.140 |
| MLBP | 0.757 | 0.744 | 0.736 | 0.706 | 0.093 |
| MPMABP | 0.741 | 0.739 | 0.722 | 0.688 | 0.099 |
| iMFP-LG | 0.797 | 0.803 | 0.796 | 0.788 | 0.078 |

*Note*: **↑** means a larger value is better on this metric; **↓** means a smaller value is better on this metric.
