# Supplementary material for: iMFP-LG: Identify Novel Multi-functional Peptides Using Protein Language Models and Graph-based Deep Learning
Source: Genomics Proteomics Bioinformatics. 2024 Nov 25;22(6):qzae084. doi: 10.1093/gpbjnl/qzae084 (PMC12011362; doi:10.1093/gpbjnl/qzae084)
Supplement: qzae084_Supplementary_Data [file qzae084_supplementary_data.zip › Table S7.docx]

**Table S7 The settings of STREME for finding the motif of AMP in MFBP dataset**

| **Parameter** | **value** |
| --- | --- |
| Minimum width | 3 |
| Maximum width | 6 |
| *P* value threshold | 0.005 |
| Number of motifs | 10.000 |
| Align sequences on their | Centers |
