# Supplementary material for: iMFP-LG: Identify Novel Multi-functional Peptides Using Protein Language Models and Graph-based Deep Learning
Source: Genomics Proteomics Bioinformatics. 2024 Nov 25;22(6):qzae084. doi: 10.1093/gpbjnl/qzae084 (PMC12011362; doi:10.1093/gpbjnl/qzae084)
Supplement: qzae084_Supplementary_Data [file qzae084_supplementary_data.zip › Table S5.docx]

**Table S5 The performance of the MFBP experiment model with 10 repetitions on MFBP test set**

| **Model** | **Precision ↑** | **Coverage ↑** | **Accuracy ↑** | **Absolute true ↑** | **Absolute false ↓** |
| --- | --- | --- | --- | --- | --- |
| Model0 | 0.778 | 0.792 | 0.777 | 0.761 | 0.090 |
| Model1 | 0.789 | 0.805 | 0.788 | 0.769 | 0.085 |
| Model2 | 0.774 | 0.784 | 0.773 | 0.760 | 0.089 |
| Model3 | 0.770 | 0.776 | 0.769 | 0.762 | 0.091 |
| Model4 | 0.776 | 0.787 | 0.775 | 0.761 | 0.090 |
| Model5 | 0.758 | 0.767 | 0.757 | 0.747 | 0.094 |
| Model6 | 0.788 | 0.801 | 0.787 | 0.772 | 0.085 |
| Model7 | 0.776 | 0.786 | 0.774 | 0.761 | 0.090 |
| Model8 | 0.773 | 0.783 | 0.771 | 0.759 | 0.091 |
| Model9 | 0.761 | 0.768 | 0.760 | 0.751 | 0.092 |
| Model_avg_ | 0.797 | 0.803 | 0.796 | 0.788 | 0.078 |

*Note*: ↑ means a larger value is better on this metric; ↓ means a smaller value is better on this metric. Model0-9 mean the results of the model repeated 10 times with different random seeds. Model_avg_ means averaged the results of 10 model (Model0-9) predictions as the final prediction for testing samples.
