# Supplementary material for: iMFP-LG: Identify Novel Multi-functional Peptides Using Protein Language Models and Graph-based Deep Learning
Source: Genomics Proteomics Bioinformatics. 2024 Nov 25;22(6):qzae084. doi: 10.1093/gpbjnl/qzae084 (PMC12011362; doi:10.1093/gpbjnl/qzae084)
Supplement: qzae084_Supplementary_Data [file qzae084_supplementary_data.zip › Table S2.docx]

**Table S2 The performance comparison of different feature extraction methods with and without GAT on MFTP dataset**

| **Model** | **Precision ↑** | **Coverage ↑** | **Accuracy ↑** | **Absolute true ↑** | **Absolute false ↓** |
| --- | --- | --- | --- | --- | --- |
| CF | 0.362 | 0.333 | 0.323 | 0.283 | 0.047 |
| CF with GAT | 0.421 | 0.408 | 0.381 | 0.324 | 0.049 |
| CNN | 0.615 | 0.603 | 0.572 | 0.509 | 0.037 |
| CNN with GAT | 0.637 | 0.626 | 0.592 | 0.525 | 0.037 |
| RNN | 0.624 | 0.614 | 0.584 | 0.522 | 0.035 |
| RNN with GAT | 0.653 | 0.645 | 0.610 | 0.542 | 0.035 |
| CNN-BiLSTM | 0.661 | 0.650 | 0.616 | 0.546 | 0.033 |
| CNN-BiLSTM with GAT | 0.680 | 0.671 | 0.635 | 0.564 | 0.034 |
| pLM | 0.709 | 0.705 | 0.667 | 0.595 | 0.032 |
| pLM with GAT | 0.721 | 0.722 | 0.697 | 0.605 | 0.032 |

*Note*: **↑** means a larger value is better on this metric; **↓** means a smaller value is better on this metric.
