# Supplementary material for: iMFP-LG: Identify Novel Multi-functional Peptides Using Protein Language Models and Graph-based Deep Learning
Source: Genomics Proteomics Bioinformatics. 2024 Nov 25;22(6):qzae084. doi: 10.1093/gpbjnl/qzae084 (PMC12011362; doi:10.1093/gpbjnl/qzae084)
Supplement: qzae084_Supplementary_Data [file qzae084_supplementary_data.zip › Table S1.docx]

**Table S1 The performance comparison of different feature extraction methods with and without GAT on MFBP dataset**

| **Model** | **Precision ↑** | **Coverage ↑** | **Accuracy ↑** | **Absolute true ↑** | **Absolute false ↓** |
| --- | --- | --- | --- | --- | --- |
| CF | 0.623 | 0.619 | 0.606 | 0.576 | 0.118 |
| CF with GAT | 0.667 | 0.649 | 0.646 | 0.623 | 0.121 |
| CNN | 0.688 | 0.706 | 0.687 | 0.667 | 0.108 |
| CNN with GAT | 0.726 | 0.745 | 0.726 | 0.706 | 0.102 |
| RNN | 0.741 | 0.747 | 0.732 | 0.709 | 0.094 |
| RNN with GAT | 0.759 | 0.763 | 0.747 | 0.719 | 0.094 |
| CNN-BiLSTM | 0.735 | 0.749 | 0.734 | 0.718 | 0.095 |
| CNN-BiLSTM with GAT | 0.761 | 0.775 | 0.757 | 0.735 | 0.091 |
| pLM | 0.774 | 0.784 | 0.774 | 0.762 | 0.082 |
| pLM with GAT | 0.777 | 0.785 | 0.776 | 0.767 | 0.082 |

*Note*: **↑** means a larger value is better on this metric; **↓** means a smaller value is better on this metric.
