# Supplementary figures and images for: iMFP-LG: Identify Novel Multi-functional Peptides Using Protein Language Models and Graph-based Deep Learning
Source: Genomics Proteomics Bioinformatics. 2024 Nov 25;22(6):qzae084. doi: 10.1093/gpbjnl/qzae084 (PMC12011362; doi:10.1093/gpbjnl/qzae084)

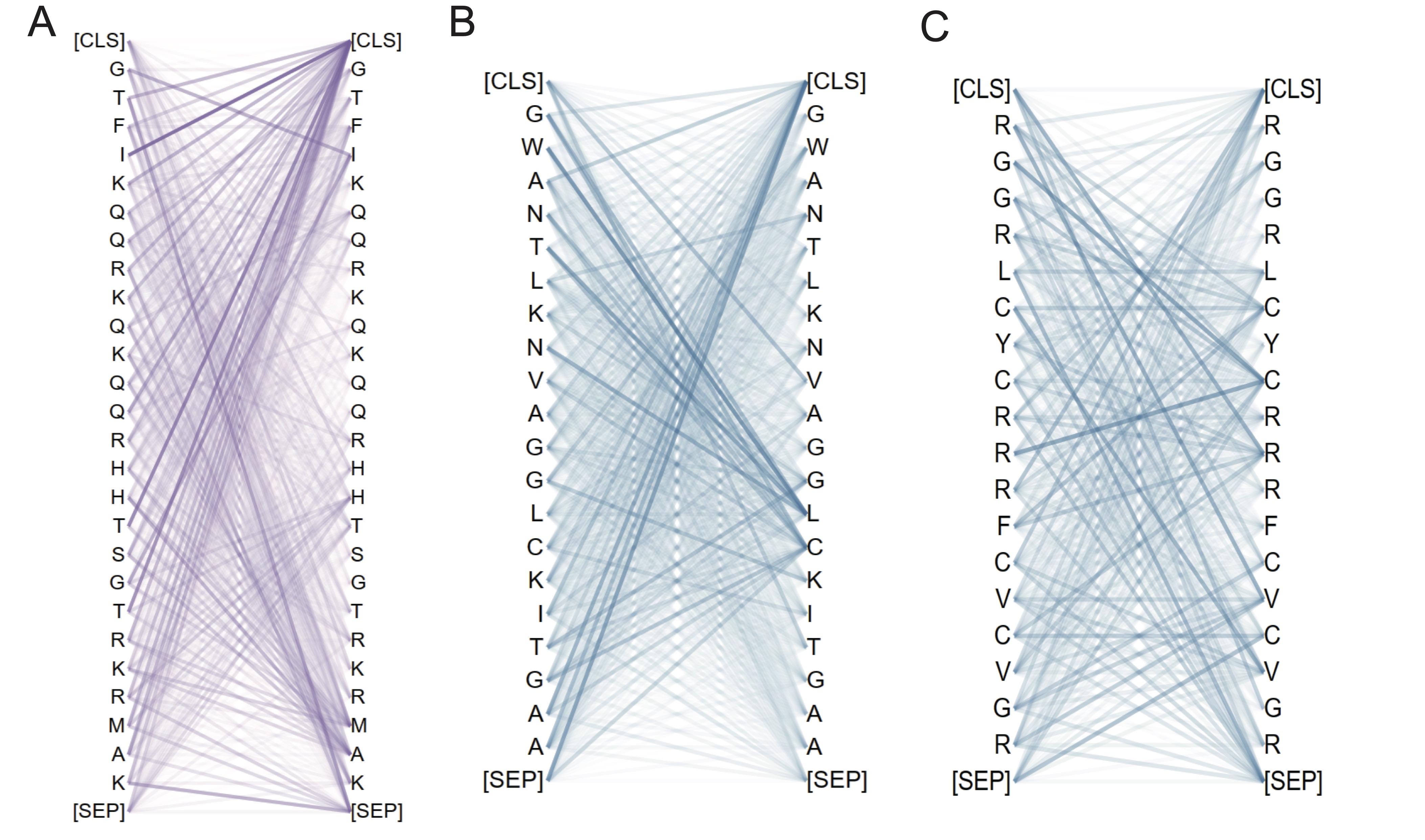

Supplement: qzae084_Supplementary_Data [file qzae084_supplementary_data.zip › Figure S1.jpg]

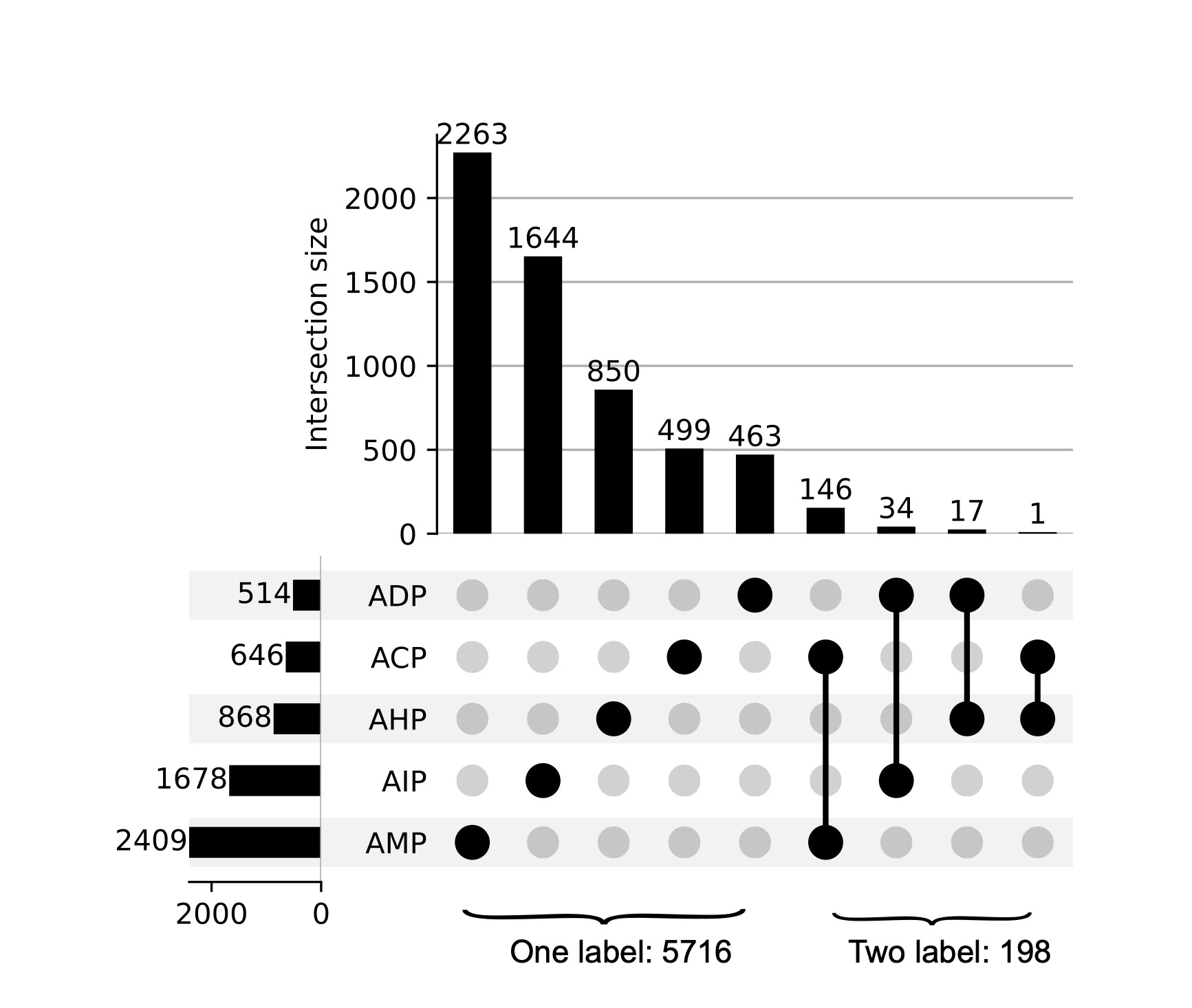

Supplement: qzae084_Supplementary_Data [file qzae084_supplementary_data.zip › Figure S3.jpg]

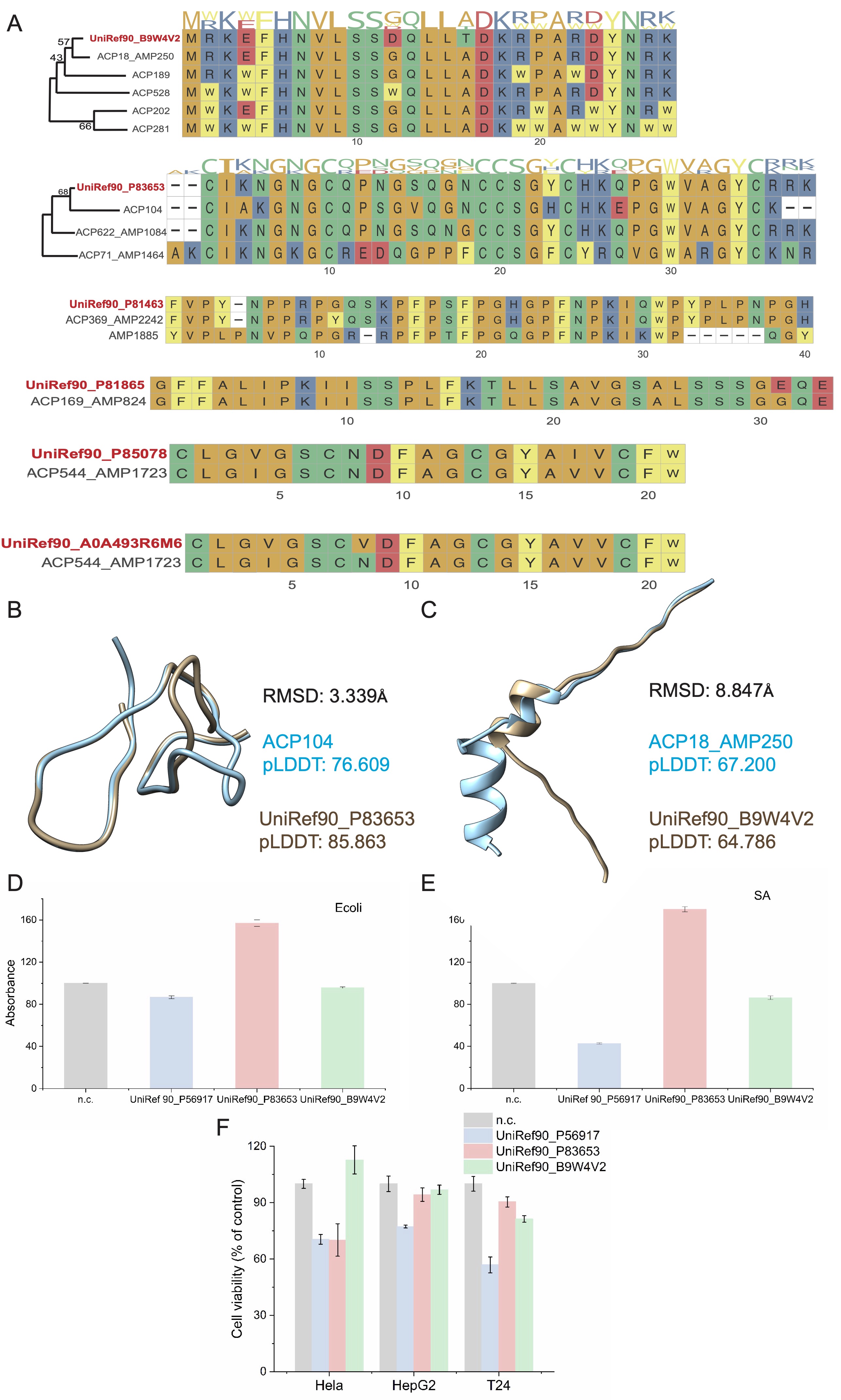

Supplement: qzae084_Supplementary_Data [file qzae084_supplementary_data.zip › Figure S2.jpg]
